# Dynamics of Type I and Type II Interferon Signature Determines Responsiveness to Anti-TNF Therapy in Rheumatoid Arthritis

**DOI:** 10.3389/fimmu.2022.901437

**Published:** 2022-06-06

**Authors:** Takeshi Iwasaki, Ryu Watanabe, Hiromu Ito, Takayuki Fujii, Kenji Okuma, Takuma Oku, Yoshitaka Hirayama, Koichiro Ohmura, Koichi Murata, Kosaku Murakami, Hiroyuki Yoshitomi, Masao Tanaka, Shuichi Matsuda, Fumihiko Matsuda, Akio Morinobu, Motomu Hashimoto

**Affiliations:** ^1^ Department of Rheumatology and Clinical Immunology, Kyoto University Graduate School of Medicine, Kyoto, Japan; ^2^ Center for Genomic Medicine, Kyoto University Graduate School of Medicine, Kyoto, Japan; ^3^ Department of Advanced Medicine for Rheumatic Diseases, Kyoto University Graduate School of Medicine, Kyoto, Japan; ^4^ Department of Clinical Immunology, Osaka Metropolitan University Graduate School of Medicine, Osaka, Japan; ^5^ Department of Orthopaedic Surgery, Kurashiki Central Hospital, Okayama, Japan; ^6^ Center for Innovation in Immunoregulative Technology and Therapeutics, Kyoto University Graduate School of Medicine, Kyoto, Japan; ^7^ Candidate Discovery Science Labs, Astellas Pharma Inc., Ibaraki, Japan; ^8^ Department of Orthopaedic Surgery, Kyoto University Graduate School of Medicine, Kyoto, Japan; ^9^ Department of Immunology, Kyoto University Graduate School of Medicine, Kyoto, Japan

**Keywords:** anti-TNF therapy, multi-omics analysis, rheumatoid arthritis, type I interferon signature, type II interferon signature

## Abstract

The factors influencing long-term responses to a tumor necrosis factor inhibitor (TNFi) in rheumatoid arthritis (RA) patients currently remain unknown. Therefore, we herein conducted a multi-omics analysis of TNFi responses in a Japanese RA cohort. Blood samples were collected from 27 biological disease-modifying antirheumatic drug (DMARD)-naive RA patients at the initiation of and after three months of treatment with TNFi. Treatment responses were evaluated at one year. Differences in gene expression levels in peripheral blood mononuclear cells (PBMCs), plasma protein levels, drug concentrations, and the presence/absence of anti-drug antibodies were investigated, and a cell phenotypic analysis of PBMCs was performed using flow cytometry. After one year of treatment, thirteen patients achieved clinical remission (responders), while the others did not or switched to other biologics (non-responders). Differentially expressed genes related to treatment responses were enriched for the interferon (IFN) pathway. The expression of type I IFN signaling-related genes was higher in non-responders than in responders before and after treatment (*P* = 0.03, 0.005, respectively). The expression of type II IFN signaling-related genes did not significantly differ before treatment; however, it increased in non-responders and decreased in responders, with a significant difference being observed after three months of treatment (*P* = 1.2×10^-3^). The total number of lymphocytes and C-X-C Motif Chemokine Ligand 10 (CXCL10) protein levels were associated with the type I IFN signature (*P* = 6.7×10^-7^, 6.4×10^-3^, respectively). Hepatocyte growth factor (HGF) protein levels before treatment predicted fold increases in type II IFN (*P* = 0.03). These IFN signature-related indices (the number of lymphocytes, CXCL10, and HGF) significantly differed between responders and non-responders (*P* = 0.01, 0.01, and 0.04, respectively). A single-cell analysis revealed that the type I IFN signature was more highly enriched in monocytes than in other cell types. A deconvolution analysis of bulk-RNA sequence data identified CD4+ and CD8+ T cells as the main sources of the type II IFN signature in non-responders. Collectively, the present results demonstrated that the dynamics of the type I and II IFN pathways affected long-term responses to TNFi, providing information on its biological background and potential for clinical applications.

## Introduction

Rheumatoid arthritis (RA) is a chronic inflammatory disease that leads to progressive joint destruction (1). Clinical remission with disease-modifying antirheumatic drugs (DMARDs) in the early stages of the disease course is the key to preventing joint destruction and maintaining the quality of life (2). Some treatment options, including tumor necrosis factor inhibitor (TNFi) therapy, are now available for RA patients who do not respond to methotrexate (MTX) (2). Although 60–70% of patients show a good to moderate response to TNFi, 30–40% have no or insufficient responses (3–5). Therefore, further studies are needed to elucidate the mechanisms underlying treatment responsiveness and identify predictive biomarkers for clinical remission in patients treated with TNFi.

Several genome-wide association studies (GWAS) have so far attempted to survey susceptible genomic regions for TNFi responses (6). However, there is still no consensus about a single gene locus with a strong effect that may be replicated across multiple studies (7). Spiliopoulou et al. reported in 2019 that the ability to predict TNF responses from genotypic scores was limited (accounting for less than 1% of treatment responses) (8). These findings suggest that downstream signals, rather than a genetic predisposition, may have an important role in treatment responsiveness to TNFi.

To address this issue, dozens of omics studies, including transcriptomics, proteomics, metabolomics, and single-cell omics studies, have been conducted (6, 9, 10). However, those results have not achieved a strong consensus. Consequently, no clinically useful predictive markers have been established to date, encompassing further studies and novel perspectives. One of the limitations of these studies is the evaluation timing of drug response. Most studies have evaluated treatment responses within a relatively short period (one to three months) after treatment initiation. Since a non-negligible number of patients eventually stop responding to treatment in the long term (11), predicting long-term responses to TNFi is essential. Another limitation is an imbalance in the study population. Only a few omics studies investigated Asian populations (6). Since ethnic differences have been reported in responses to DMARDs (12), which may be attributed in part to differences in genetic polymorphisms in drug metabolism or RA pathology, further studies on Asian populations are warranted.

In the present study, we conducted a multi-omics analysis in patients who planned to initiate TNFi treatment to identify transcriptomic and proteomic features that predict responses or resistance to TNFi in RA using a multi-omics analysis of a Japanese cohort in terms of one-year efficacy.

## Materials and Methods

### Study Patients

**Figure 1:**
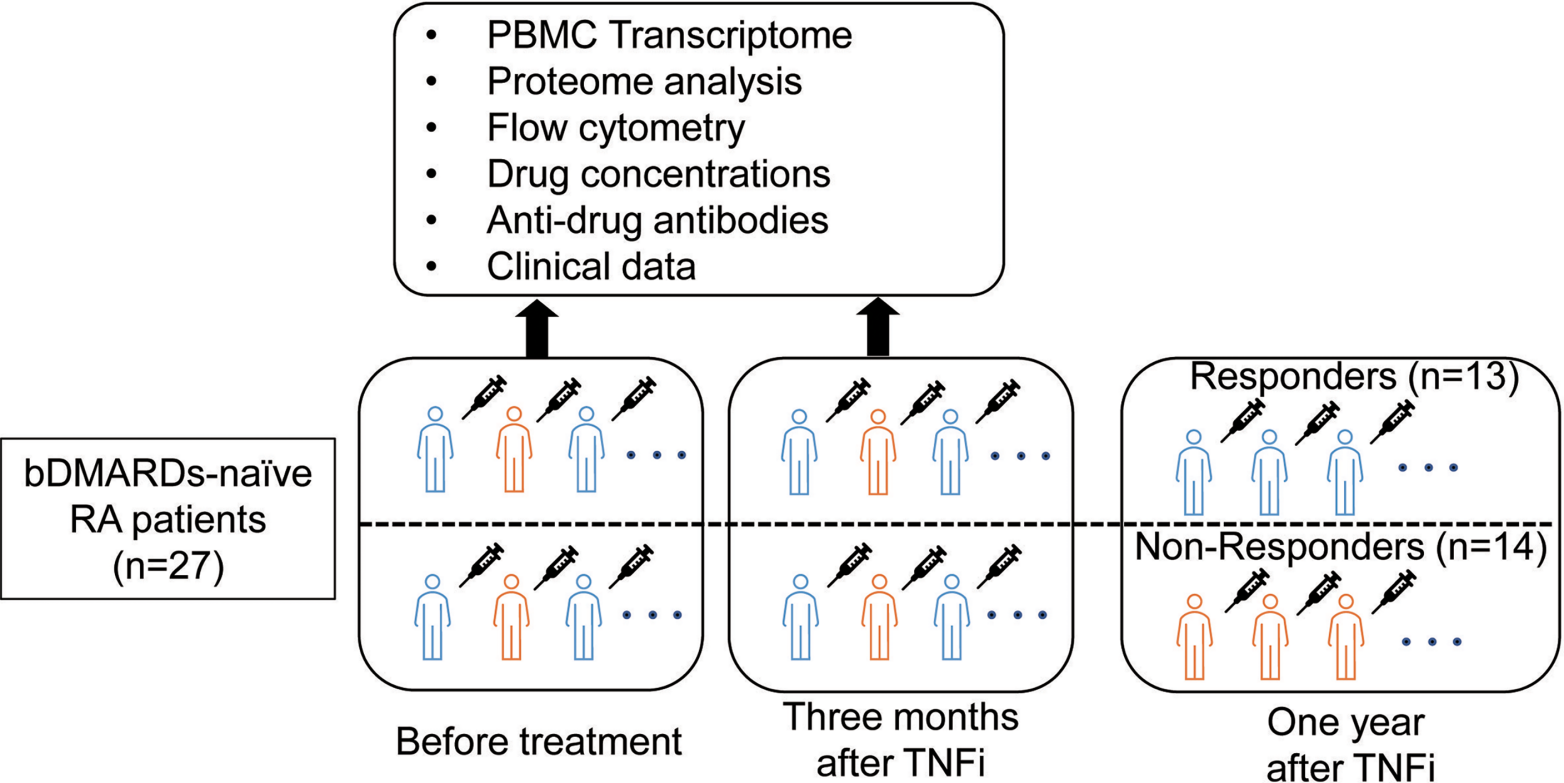
Overview of the present study.

We enrolled 29 biological DMARD-naive RA patients who were enrolled in the Kyoto University Rheumatoid Arthritis Management Alliance (KURAMA) cohort (13) and received anti-TNF therapy. The KURAMA cohort is an observational cohort that was established in May 2011 at the Center for Rheumatic Disease at Kyoto University Hospital to achieve strict control of RA. RA was diagnosed according to either the 1987 revised American College of Rheumatology classification criteria (14) or the 2010 American College of Rheumatology/European League Against Rheumatism (ACR/EULAR) criteria (15). The overview of this study is shown in **Figure 1**. Peripheral blood was collected from patients before and approximately three months after initiating TNFi treatment using heparin and ethylenediaminetetraacetic acid (EDTA) blood collection tubes. Responses to TNFi were assessed one year after the initiation of therapy.

### Clinical Evaluation

Disease activity was evaluated using the Disease Activity Score 28-Erythrocyte sedimentation rate (DAS28-ESR) at every clinic visit. Clinical characteristics, including age, sex, body mass index (BMI), smoking history, duration of RA, the titers of rheumatoid factor (RF), anti-cyclic citrullinated peptide (CCP) antibodies, anti-nuclear antibodies (ANA), ESR, C-reactive protein (CRP) and treatment profiles [the use of MTX and prednisolone (PSL)] before the initiation of treatment, and white blood cell count, blood differential count before and three months after the initiation of treatment, were obtained from medical records.

### Response Measures

We classified patients who achieved clinical remission [DAS28-ESR < 2.6 (16)] at one year as responders and those who did not or discontinued TNFi within one year due to inadequate responses as non-responders. Patients whose biologics were discontinued due to adverse events were excluded.

### Measurement of TNFi Concentrations and Anti-Drug Antibody Titers Against TNFi

In patients who received adalimumab, etanercept, or infliximab, the plasma concentration of each drug and its anti-drug antibody titer were measured before and after treatment using the assay kit SHIKARI^®^ by Matriks Biotek Laboratories. Measurements were conducted following the manufacturer’s instructions.

### RNA Sequencing and Transcriptome Analysis

Peripheral blood mononuclear cells (PBMCs) were isolated from heparin collection tubes using Lymphocyte Separation Solution (Nacalai Tesque, d=1.077, cat. 20828-15). RNA was extracted from freshly isolated PBMCs by the RNAqueous-MicroKit (Ambion, AM1931), and genomic DNA was removed with the RNase-Free DNase Set (QIAGEN, 79254). Library preparation was performed using the SMARTer Ultra low RNA Kit for Illumina Sequencing (Clontech, USA). Sequencing was conducted by HiSeq 2,500 in the 150-bp paired-end mode. Sequencing reads were trimmed using Cutadapt (ver 1.1). Trimmed FASTQ files were aligned to the GRCh37 reference genome using STAR (ver. 2.7.3a) (17). Gene counts were generated by RSEM (ver. 1.3.1) (18) using Gencode v19 (19) as a reference. Gene counts were normalized by size factor implemented in DeSeq2 (20) and converted to count per million (CPM). We conducted a principal component analysis using 13,252 expressed genes (mean CPM > 1) in 54 samples (27 patients × two-time points) and confirmed the absence of outlier samples (**Supplementary Figure 1**). The Wald test performed a gene expression analysis using DESeq2 (20). Enrichment analysis was performed by Metascape (21) with default settings.

Type I and II interferon (IFN) scores were calculated by mean expression of genes of IFN stimulated genes (22, 23) (**Supplementary Table 1**). Type I IFN-stimulated genes were defined as the union of genes included in “GOBP_RESPONSE_TO_INTERFERON_ALPHA” or “GOBP_RESPONSE_TO_INTERFERON_BETA” in the Molecular Signatures Database [MSigDB ver7.4 (24)]. Type II IFN-stimulated genes were defined as those in included in “HALLMARK_INTERFERON_GAMMA_RESPONSE” in MSigDB (ver7.4) (24).

### Flow Cytometry and Protein Measurements

The surface molecule expression was assessed using BD Canto™ II. The following antibodies were obtained from BD Pharmingen: allophycocyanin-conjugated anti-CD56 (341025, NCAM16); anti-CD3 (566683, OKT3), and V500-conjugated CD19 (561121, HIB19). We gated lymphocytes based on forward scatter (FSC) and side scatter (SSC) parameters and then calculated the percentage of each cell fraction in lymphocytes. The gating strategy is shown in **Supplementary Figure 2**. The analysis was conducted using FlowJo software. The absolute number of each cell fraction in peripheral blood (×10^6^/mL) was calculated using the percentage and an absolute number of lymphocytes measured by the hematology analyzer MEK-7300 (Nihon Kohden^®^).

We isolated plasma from peripheral blood collected in EDTA-containing tubes and measured 67 proteins using ProcartaPlex Human 15-plex, ProcartaPlex Human 49-plex, Human VCAM-1 Simplex, Human sICAM-1 Simplex, and Human sCGF Simplex with Bio-Plex 200 (BIO-RAD) according to the manufacturer’s instructions.

The analysis of the usefulness of identified blood cell phenotype and proteins for differentiating responders from non-responders used receiver-operating-characteristic (ROC) curve techniques. The calculation of area under the ROC curve (AUC) and the creation of multiple linear model using ordinary least squares (OLS) were performed using the python package sklearn (ver 0.20.4).

### Single-Cell Analysis

The cellular origin of the type I IFN signal was analyzed using single-cell data of PBMCs from RA patients (https://www.ncbi.nlm.nih.gov/geo/query/acc.cgi?acc=GSE159117). The R package Seurat (ver4.0) (25) was used for data scaling, transformation, clustering, and dimensionality reduction. The type I IFN score was calculated in the same manner as that for Bulk Transcriptome data. The scripts used are shown in **Supplementary Note**.

### Deconvolution Analysis

We performed a deconvolution analysis using CIBERSORTx (26) to estimate cell type abundance in specimens and investigate the cellular origin of the type II IFN signature, which increased during the course of treatment in non-responders. As a reference, we used the findings of bulk RNA-Seq of the sorted cell subtypes of RA patients (27). These data contain gene count data for 28 different cell subtypes of RA patients (n=24). We initially converted them to counts per million (CPM) and created a signature matrix using the CPM of 26 cell subtypes (the “fraction” function), excluding neutrophils and low-density granulocytes. As an input, we used transcripts per million (TPM) for each specimen. Since it was not possible to estimate gene expression in all 26 cell populations due to lack of statistical power, we estimated gene expression in six cell subtypes (CD4+, CD8+, natural killer cell (NK), dendritic cells (DC), and monocytes) (the “hires” and “classes” functions). To increase the statistical power of the estimation, we also included the bulk-RNA Seq data of other RA patients (n=111) processed on the same platform in the KURAMA cohort. We confirmed that the estimated cell proportion correlated with real data (T cells, B cells, and NK cells defined by CD3+, CD19+, and CD 56+, respectively) obtained from FACS data (Pearson’s correlation coefficient: 0.87). We calculated the mean expression levels of the estimated type II IFN-related genes, excluding missing values, and calculated fold changes in each cell fraction after three months of treatment from before treatment. Differences in fold changes between responders and non-responders were tested by the Mann-Whitney U test.

### Ethics Approval and Consent to Participate

The present study was performed in accordance with the Helsinki Declaration and was approved by the Kyoto University Graduate School and Faculty of Medicine Ethics Committee (approval number: G0511-6). Written informed consent to participate in the present study and publish the results obtained was provided by all enrolled patients.

## Results

### Comparison of Clinical Characteristics of Responders and Non-Responders to TNFi

Twenty-nine biological DMARD-naïve RA patients were registered from the KURAMA cohort. Two patients discontinued the use of TNFi because of an injection site reaction (7 and 10 months after treatment initiation, respectively); therefore, 27 patients were enrolled in the present study. After one year of treatment, 13 (48.1%) patients received the same TNFi and achieved remission, while 14 (51.9%) did not achieve remission or TNFi was switched to other agents. The clinical characteristics of patients are summarized in **Table 1**. ESR was significantly higher in non-responders than in responders (*P* = 0.02) before treatment. CRP and DAS28-ESR before treatment were slightly higher in non-responders (*P* = 0.054 and *P* = 0.08, respectively). No significant differences were observed in age, sex, BMI, smoking history, duration of RA, RF, anti-CCP, ANA, or the use of MTX and PSL between responders and non-responders (*P* > 0.05).

**Table 1:** Clinical characteristics of study patients.

		Responders	Non-responders	OR (95% CI)	P*
Total		13	14		
Age		66.0 (60.0-68.0)	61.5 (56.8-69.8)	ND	0.85
Female (%)		9 (69.2)	11 (78.6)	1.6 (0.2-14.0)	0.68
BMI		21.1 (19.5-23.1)	20.7 (19.6-24.1)	ND	0.98
Smoking history (%)		2 (22.2)	4 (33)	1.7 (0.18-24.4)	0.66
Duration of RA (years)		1.6 (0.6-6.4)	1.6 (1.2-6.6)	ND	0.94
CRP (mg/dL)		0.4 (0.1-0.9)	1.35 (0.5-4.2)	ND	0.054
ESR (mm/h)		23.0 (14.0-38.0)	47.5 (25.8-67.0)	ND	0.02
RF titer (U/mL)		24.8 (9.9-38.6)	71.8 (17.0-126.3)	ND	0.33
Anti-CCP positivity (%)		9 (69.2)	10 (71.4)	1.1 (0.2-8.0)	1
Anti-nuclear antibodies (≥80)		2 (15.4)	3 (21.4)	1.5 (0.14-21.0)	1
MTX usage (%)		12 (92.3)	14 (100.0)	Inf (0.03-Inf)	0.48
MTX dosage^†^ (mg/week)		8.0 (6.0-10.0)	8.0 (8.0-10.0)	ND	0.31
PSL usage (%)		3 (23.1)	3 (21.4)	0.9 (0.1-8.5)	1
PSL dosage^†^ (mg/day)		0.0 (0.0-0.0)	0.0 (0.0-0.0)	ND	0.89
DAS28-ESR	0 months	3.9 (3.5-4.9)	5.0 (4.4-5.7)	ND	0.08
	3 months	2.1 (1.8-2.6)	3.4 (2.7-4.4)	ND	6.0E-03
	1 year	1.7 (1.5-2.1)	2.8 (2.6-3.5)	ND	6.2E-05
Breakdown of TNFi	Adalimumab	0 (0.0)	2 (14.3)	Inf (0.18-Inf)	0.48
	Etanercept	1 (7.7)	1 (7.1)	0.92 (0.01-78.4)	1
	Golimumab	4 (30.8)	1 (7.1)	0.18 (0.003-2.3)	0.16
	Infliximab	8 (61.5)	10 (71.4)	1.5 (0.2-10.7)	0.69
Anti-drug antibodies^††^		0 (0.0)	2 (15.4)	Inf (0.13-Inf)	0.49

Data were described as medians [interquartile range (IQR)] for continuous variables and numbers (percentages) for categorical variables. *The Mann-Whitney U test was used for continuous variables and Fisher’s exact test for categorical variables. †Calculated from all samples regardless of the corresponding drug usage. ††Tested for patients who received adalimumab, etanercept, or infliximab. OR, odds ratio; ND, no data; Inf: Infinity; BMI, Body mass index; CRP, C-reactive protein; ESR, Erythrocyte sedimentation rate; RF, rheumatoid factor; CCP, cyclic citrullinated peptide; PSL, prednisolone; MTX, methotrexate.

### Differences in Drug Concentrations, Anti-Drug Antibody Titers Between Responders and Non-Responders

Previous studies demonstrated that the efficacy of TNFi may be affected by drug concentrations and the existence of anti-drug antibodies in peripheral blood (28). To test if these previous findings can be replicated, we examined the concentrations of TNFi and anti-drug antibody titers against TNFi. We confirmed that the corresponding drug concentrations were below the detection level in all samples before treatment. After treatment, no significant differences were noted in drug concentrations between responders and non-responders (**Supplementary Table 2**). The anti-drug antibodies of the corresponding TNFi were not detected in patients before treatment but were found in 2 non-responders (15%) after treatment. In contrast, none of the responders had anti-drug antibodies (Fisher’s exact test, *P* = 0.49, **Table 1**).

### Relationship Between Gene Expression and Responses to TNFi

**Figure 2:**
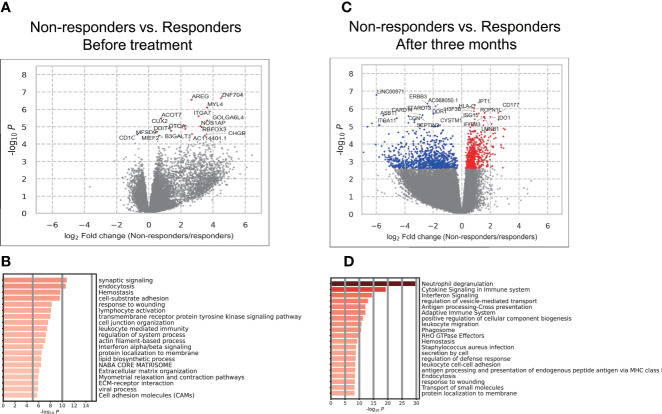
Results of the association analysis of gene expression. **(A, C)** Volcano plots showing differentially expressed genes (DEGs) in PBMCs between responders and non-responders before treatment **(A)**. and three months after **(C)**. Each dot indicates an individual gene, colored in red when a gene was significantly (FDR < 0.05) up-regulated in non-responders and colored in blue when a gene was significantly (FDR < 0.05) up-regulated in responders. The gene names of significant DEGs are shown; when there were more than 20 significant DEGs, the names of only the top 20 DEGs were shown. **(B, D)** The results of the enrichment analysis of DEGs before treatment **(B)** and three months after **(D)**.

We conducted a differential gene expression analysis between responders and non-responders before treatment. We identified 17 genes associated with treatment responses (False discovery rate; FDR < 0.05) (**Figure 2A**, **Supplementary Table 3**). To characterize differentially expressed genes, we conducted an over-representation analysis using MetaScape (21). Since the number of genes with FDR < 0.05 was too small, we used the 2,678 genes that showed nominal significance (*P* < 0.05). As a result, we found that type I IFN signaling placed 12^th^ in the top enriched pathways (**Figure 2B**, **Supplementary Table 4**).

We also conducted a differential gene expression analysis between responders and non-responders after treatment. Among the 17 genes which showed a significant difference between responders and non-responders, three genes (MYL4, ACOT7, DDIT4) remained higher in non-responders with significance. The remaining 14 genes lost their significance or showed associations in the opposite directions. Overall, significant differences were observed in the expression of 1,469 genes (FDR < 0.05) (**Figure 2C**, **Supplementary Table 5**). To characterize these genes, we conducted an enrichment analysis. IFN signaling placed 3^rd^ (**Figure 2D**) and the type I and II IFN signaling pathways were enriched (**Supplementary Table 6**). These results implicated the type I and II IFN pathways in responses to TNFi in RA patients. Although the involvement of type I IFN in short-term responses has been demonstrated (29–32), its contribution to long-term efficacy and the role of type II IFN have not yet been examined. Therefore, these results prompted us to investigate its involvement in TNFi responses in more detail.

### Dynamics of IFN Signatures Related to TNFi Responses

**Figure 3:**
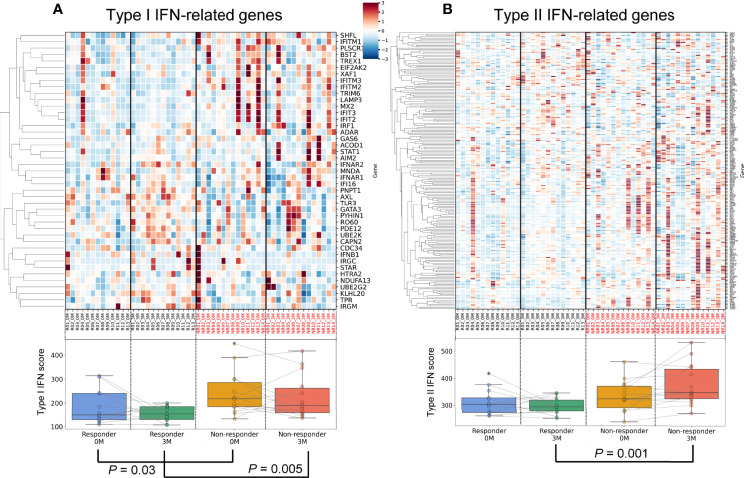
Differences in and dynamics of type I and II interferon signatures. An upper heatmap shows the expression of type I IFN-related genes **(A)** and type II IFN-related genes **(B)**. The expression of each gene (CPM) related to the type I IFN signature was standardized across all samples, and Z scores were shown. Lower box plots show the type I IFN score **(A)** and type II IFN score **(B)** for each specimen.

The IFN signature is represented as the IFN score. We calculated the IFN score using the average expression of IFN-stimulated genes (22, 23) in each specimen (**Figure 3**). The results obtained revealed that the type I IFN score was significantly higher in non-responders before treatment (*P* = 0.03) and three months after treatment (*P* = 0.005) (**Figure 3A**, **Supplementary Table 7**). These results indicated that the presence of the type I IFN signature before treatment was associated with the poor therapeutic efficacy of TNFi, which is consistent with previous findings (29).

Regarding type II IFN, no significant differences were observed between responders and non-responders before treatment (*P* = 0.11, **Figure 3B**, **Supplementary Table 7**). During treatment, type II IFN scores slightly increased in non-responders (*P* = 0.10), but slightly decreased in responders (*P* = 0.29). After three months of treatment, a significant difference was observed in type II IFN scores between responders and non-responders (*P* = 0.001). Collectively, transcriptomic data demonstrated that type I IFN scores to TNFi were persistently high in non-responders, while a post-treatment increase was observed in type II IFN scores. These results highlighted the contrasting dynamics of the type I and type II IFN signatures between TNFi responders and non-responders.

### Identifying Alternative Biomarkers of the IFN Signature

We demonstrated the utility of IFN scores for predicting treatment responses. Since the transcriptome is generally unavailable and difficult to adopt in daily clinical practice, we examined alternative markers by investigating the relationships between IFN scores and various traits.

**Figure 4:**
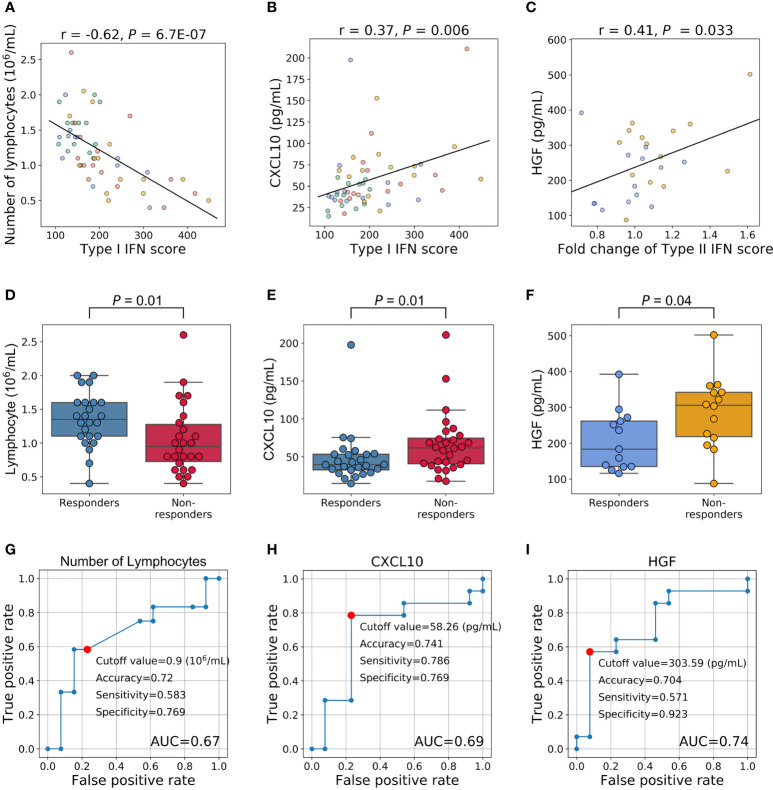
Relationships between the IFN signature and other phenotypes and an evaluation of their clinical utility as predictive markers. **(A–C)** Relationships between IFN scores and other phenotypes. Each dot represents each specimen. Blue represents responders before treatment, orange for non-responders before treatment, green for responders three months after treatment, and red for non-responders three months after treatment. **(D, E)** Relationships between treatment responses and the number of lymphocytes and CXCL10 levels. Fifty-four specimens (27 samples × 2 timepoints) were analyzed. **(F)** Relationship between HGF levels before treatment and treatment responses. **(G–I)** ROC curves for no response to TNFi. Red plots indicate the cut-off points at the highest accuracy (minimum false negative and false positive results) for predicting non-responders.

We initially examined the relationships between type I IFN scores and various traits using 54 specimens (27 samples × 2 time points). Type I IFN scores did not correlate with DAS28-ESR and each component (**Supplementary Figures 3A–E**, *P* > 0.05). We then investigated the relationship between type I IFN scores and the number of cells of each subpopulation. A strong negative correlation was noted between type I IFN scores and the number of lymphocytes (**Figure 4A**, *P* = 6.7E-07). This correlation was observed across each cell fraction of PBMCs (CD3+, CD19+, CD56+; *P* = 1.6×10^-5^, 3.3×10^-5^, 4.3×10^-3^, respectively) (**Supplementary Table 8**). We confirmed that non-responders had slightly lower lymphocyte counts both before (**Supplementary Figure 4A**) and after treatment (**Supplementary Figure 4B**). When we combined the results obtained, a significant difference was noted (*P* = 0.01, **Figure 4D**). We also examined relationships with 67 proteins measured in our multi-omics platform. While positive correlations (Pearson’s coefficient > 0) were observed with the proteins IFNα and IFNβ (**Supplementary Table 9**), the protein with the strongest correlation was C-X-C Motif Chemokine Ligand 10 (CXCL10) (**Figure 4B**). Although CXCL10 is generally known as a type II IFN-inducible protein, its expression was also shown to be induced by type I IFN (33). CXCL10 levels were high in non-responders before (**Supplementary Figure 4C**) and after three months (**Supplementary Figure 4D**) of treatment. When we combined results, a significant difference was observed (*P* = 0.01, **Figure 4E**). Therefore, type I IFN-associated indices appear to be useful for distinguishing non-responders from responders.

We also investigated whether pre-treatment traits predicted increases in the type II IFN score. To achieve this, we assessed the relationships between pre-treatment traits and fold changes in type II IFN scores (the type II IFN score after 3 months divided by the score before treatment). A correlation was not observed with DAS28-ESR and each component(*P* > 0.05, **Supplementary Figures 3F–J**). Furthermore, no significant predictive markers were identified for blood cell counts (**Supplementary Table 10**). However, among the proteins examined, a correlation was noted with the protein levels of hepatocyte growth factor (HGF) (*P* = 0.03, **Figure 4C**, **Supplementary Table 11**). HGF levels were higher in non-responders before treatment (*P* = 0.04, **Figure 4F**).

**Figure 5:**
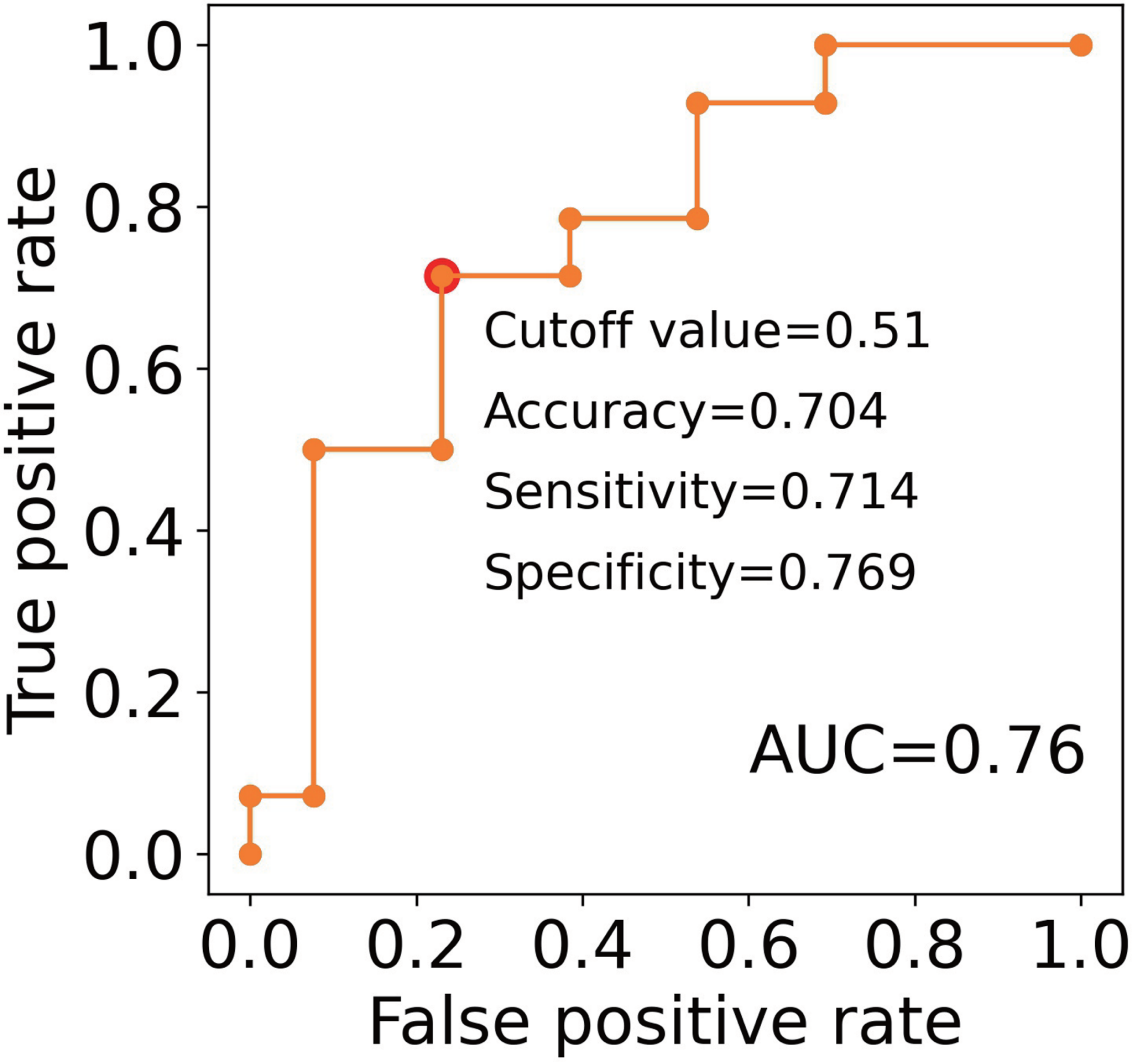
An evaluation of the clinical utility of the combinations of predictive markers. ROC curves for non-response to TNFi. The model created by the multiple linear regression was as follows; 2.0×10^-3^ × HGF (pg/mL) + 6.2×10^-4^×CXCL10 (pg/mL) - 0.13×Number of lymphocytes (10^6^/μL) + 0.13. Red plots indicate the cut-off points at the highest accuracy (minimum false negative and false positive results) for predicting non-responders.

These results support the potential to predict the clinical outcomes of RA patients treated with TNFi by measuring the number of lymphocytes and the levels of CXCL10 and HGF before treatment initiation. To confirm this hypothesis, we performed a ROC analysis. AUC for these three proteins were 0.67, 0.69, and 0.74, respectively (**Figures 4G–I**). The most accurate cut-off levels were 0.9×10^6^/mL, 58.26 pg/mL, and 303.59 pg/mL, which had accuracies of 72.0, 74.1, and 70.4%, sensitivities of 58.3, 78.6, and 57.1%, specificities of 76.9, 76.9, and 92.3%, respectively. We have evaluated the proportion of responders and non-responders based on the cut-off values by the χ2 test and obtained significance for HGF and CXCL10 (*P* = 0.02, 0.01, respectively) and suggestive significance for the number of lymphocytes (*P* = 0.06). Furthermore, these three IFN-signature-related indices showed some degree of independence from each other (|r| < 0.4, **Supplementary Figure 5**). Motivated by this, we finally created multiple linear regression model (2.0×10^-3^×HGF (pg/mL) + 6.2×10^-4^×CXCL10 (pg/mL) - 0.13×Number of lymphocytes (10^6^/μL) + 0.13), and obtained higher prediction accuracy (AUC = 0.76, **Figure 5**). Collectively, these results suggest that the IFN signature and its related phenotype are good predictors of responsiveness to TNFi.

### Cellular Origin of the IFN Signature

**Figure 6:**
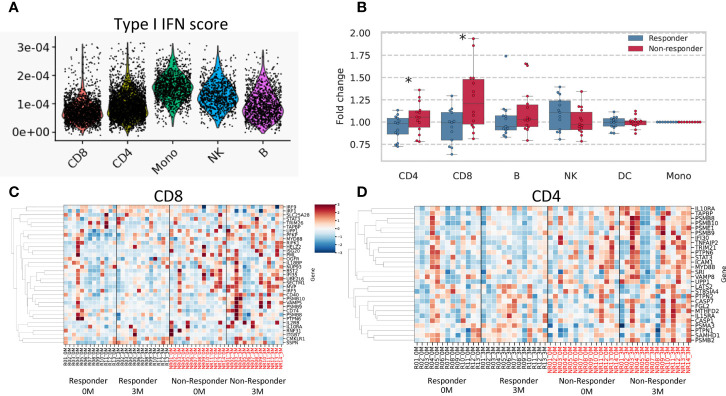
Results of single-cell data and a deconvolution analysis of the IFN signature. **(A)** Type I IFN scores for each cell subtype are shown. **(B)** Fold changes (after/before treatment) in the mean expression of type II IFN-related genes are shown according to each cell subtype. A heatmap of estimated expression in cells positive for CD8 **(C)** and CD4 **(D)**. The estimated expression of each gene was standardized across all samples, and Z scores were shown. * stands for *p* < 0.05 by the Mann-Whitney U test. CD4, CD4-positive cells; CD8, CD8-positive cells; B, B cells; DC, Dendritic cells; NK, Natural killer cells.

To identify the cellular origin of the type I and II IFN signatures uniquely detected in non-responders, we analyzed the open-access single-cell data of PBMC in RA patients. Although the type I IFN signature was ubiquitously observed across each cell type, its abundance was significantly higher in monocytes (monocytes vs. CD4+, CD8+, B, NK cells: *P* < 2.2×10^-6^, respectively; **Figure 6A**).

We conducted a deconvolution analysis to dissect heterogenous PBMC transcriptomes and estimate cell type-specific type II IFN-related gene expression in each specimen using CIBERSORTx (26). We compared fold increases in type II IFN scores between responders and non-responders for each cell type. The results obtained showed that fold changes in type II IFN scores were higher in non-responders than in responders for CD4+ and CD8+ cells (*P* = 0.047, 0.01, respectively) (**Figures 6B–D**). No significant differences were observed for other cell types. These results suggested that T cells were responsible for the non-responder-specific increase in the type II IFN signature after treatment.

## Discussion

In the present study, a transcriptome analysis showed that a persistently high type I IFN signature and a post-treatment increase in the type II IFN signature were features that were unique to non-responders. The number of lymphocytes and the level of CXCL10 were associated with the type I IFN signature, while the level of HGF before treatment was associated with fold changes in the type II IFN signature after the TNFi treatment. Consistent with these results, non-responders had a lower lymphocyte count and higher levels of CXCL10 and HGF before treatment. Collectively, the present results demonstrated that the dynamics of the type I and II IFN signatures affected long-term responsiveness to TNFi and that these three indices have potential as alternative biomarkers of the type I and type II IFN signatures in TNFi-treated patients.

To the best of our knowledge, this is the first study to report the involvement of the type II IFN signature in responses to TNFi. Although TNF was previously shown to regulate type I IFN (34, 35), the present results indicated that it also regulated type II IFN. The results of the deconvolution analysis suggested that an elevated type II IFN response signature was derived from T cells. Therefore, T cells activated by type II IFN may be a potential treatment target in TNFi non-responders. These results support the inhibition of Janus kinase, the downstream signal of the IFN pathway (36), being a good therapeutic option when TNFi fails.

We found a strong negative correlation between the blood type I IFN signature and lymphocytes. A previous study reported that the treatment of chronic hepatitis C patients with IFN alpha exerted suppressive effects on hematopoiesis (37). Therefore, the present results suggested that the internal type I IFN signature in RA patients also exhibited anti-proliferative activity against lymphocytes.

CXCL10, one of the IFN-inducible proteins, showed the strongest correlation with the type I IFN signature, and higher levels of CXCL10 predicted a poor response in the present study. A pathogenic role for CXCL10 in arthritis was previously demonstrated in RA patients (38) as well as in mouse models (39). Based on these findings and our results, the IFN-CXCL10 axis and its downstream signaling may not only be a useful biomarker for TNFi resistance, but also a potential therapeutic target in TNFi treatment-resistant RA.

HGF levels, which correlated with fold changes in the type II IFN score, have been shown to promote osteoclastogenesis in mice with collagen-induced arthritis (40). Furthermore, HGF has been implicated in the progression of joint damage in RA patients (41). Although it currently remains unclear whether HGF is directly involved in increases in type II IFN, it may serve as a predictive marker of responsiveness to TNFi.

**Figure 7:**
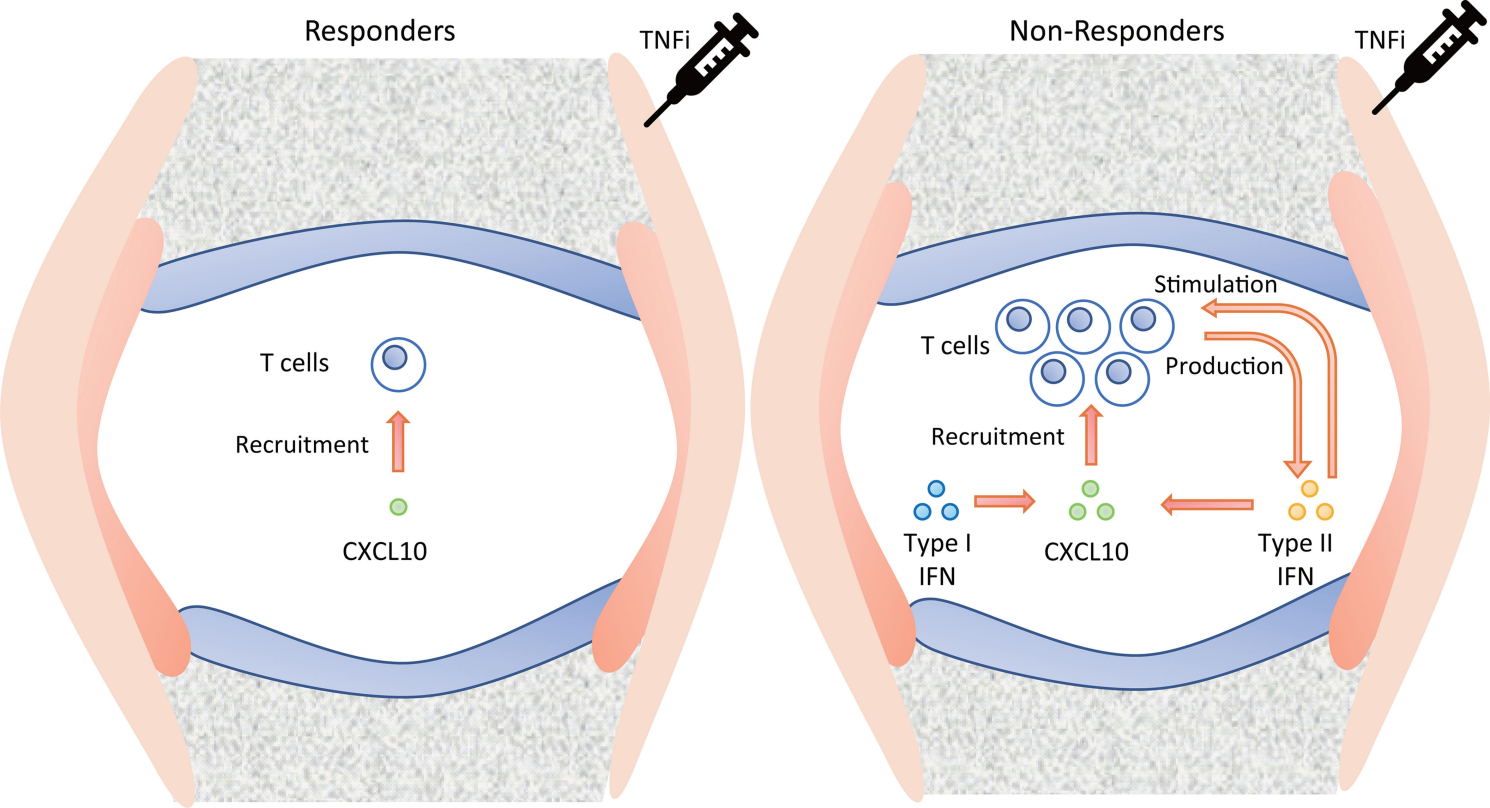
Suggested pathology of the synovium in TNFi responders and non-responders. TNFi, tumor necrosis factor inhibitor.

Our results, together with previous knowledge, suggest a possible vicious cycle in the synovium of TNFi non-responders (**Figure 7**). That is, CXCL10, which is high in non-responders, recruits T cells (42), which produce Type II IFN (43, 44). It stimulates T cells (**Figure 6B**) as well as CXCL10 production (45). CXCL10 production may also be stimulated by the Type I IFN (**Figure 4B** (33),). Therefore, abatacept, which targets T cells, might be an important candidate as alternative therapy when TNFi treatment fails.

The findings of some studies are consistent with the present results, whereas others are not. The relationship observed between the high type I IFN signature before treatment and poor responses in the present study is consistent with previous findings (29, 30). On the other hand, elevated IFN signaling in neutrophils has been identified as a favorable therapeutic response (31, 32). This discrepancy may be explained as follows: 1) the timing of evaluations, with previous studies examining treatment responses at three months; 2) cell populations, with previous studies investigating the transcriptome of neutrophils; and 3) ethnicities, with previous studies enrolling Europeans. Moreover, the reported relationships between good responses and a high neutrophil-to-lymphocyte ratio (46) as well as higher levels of CXCL10 (47) are in contrast to the present results; however, the EULAR criteria were used to distinguish responders from non-responders in these studies. Since the EULAR response criteria define treatment response by ΔDAS28, patients with high disease activity might be classified as good responders. Such patients would eventually be switched to a different biologic in the long term and would be classified as non-responders by our definition. Indeed, in our study, among 13 patients who had a good response on EULAR after 3 months, approximately half (6 patients) were classified as non-responders in our definition. This drastic phenotypic change due to differences in classification algorithms might be the main reason for the discrepancy between our study and previous reports. One of the limitations of the present study is its relatively small sample size. Therefore, the results obtained need to be validated in other cohorts. Nevertheless, to the best of our knowledge, this is the first study to report long-term responses to TNFi, which provides biological insights into TNFi responses and potential therapeutic strategies for RA.

## Data Availability Statement

All normalized gene counts for this study can be found in public, open access repository [ImmPort https://www.immport.org/shared/home (48)] with the accession ID SDY1924. All proteins, FACS data and table of correspondence between sample IDs in ImmPort and IDs in this paper are included in the **Supplementary Table 12**. Further data relating to the experiments described in this paper is available upon request; please contact the corresponding author.

## Ethics Statement

The studies involving human participants were reviewed and approved by The Kyoto University Graduate School and Faculty of Medicine Ethics Committee (approval number: G0511-6). The patients/participants provided their written informed consent to participate in this study.

## Author Contributions

TI wrote the first draft of the manuscript. RW revised and finalized the manuscript. All authors were involved in drafting the article or revising it critically for important intellectual content, and all authors approved the final version to be published. TI, RW, HI, MH contributed to the study conception and design. TI, RW, HI, KOk, TO, YH, KOh, KMurat, KMurak, MT, SM, AM, MH contributed to the acquisition of data. TI, RW, TF, KOh, HY, FM, MH contributed to analysis and/or interpretation of data.

## Funding

This study received funding in part from the Special Coordination Funds for Promoting Science and Technology of the Japanese Government. The Department of Advanced Medicine for Rheumatic Diseases is supported by Nagahama City, Shiga, Japan, Toyooka City, Hyogo, Japan, and five pharmaceutical companies (Mitsubishi Tanabe Pharma Co., Chugai Pharmaceutical Co. Ltd, UCB Japan Co. Ltd, AYUMI Pharmaceutical Co., and Asahi Kasei Pharma Corp.). The KURAMA cohort is also supported by grant from Daiichi Sankyo Co. Ltd. The funders were not involved in the study design, collection, analysis, interpretation of data, the writing of this article or the decision to submit it for publication.

## Conflict of Interest

RW received speaker fees from Mitsubishi Tanabe Pharma, Pfizer, Sanofi, AbbVie, Asahi Kasei, Eisai, Eli Lilly, Bristol-Myers Squibb, and Janssen. KOk, TO, YH are employees of Astellas Pharma Inc. KOh received research grants and/or speaker’s fees from Abbvie, Actelion, Asahikasei Pharma, Astellas, AYUMI, Bristol-Myers Squibb, Chugai, Daiichi-Sankyo, Eisai, Eli Lilly, GSK, Janssen, JB, Mitsubishi Tanabe, Nippon Kayaku, Nippon Shinyaku, Novartis, Sanofi, and Takeda. KMurat received a speaking fee from Eisai Co., Ltd., Chugai Pharmaceutical Co., Ltd.; Asahi Kasei Pharma Corp.; and Mitsubishi Tanabe Pharma Corporation. MT has received research grants and/or speaker fees from AbbVie GK, Asahi Kasei Pharma Corp., Astellas Pharma Inc., Ayumi Pharmaceutical Corp., Bristol-Myers Squibb, Chugai Pharmaceutical Co., Ltd., Eisai Co., Ltd., Eli Lilly Japan K.K., Pfizer Inc., UCB Japan Co., Ltd., Janssen Pharmaceutical K.K., Mitsubishi Tanabe Pharma Corp., Novartis Pharma K.K., Taisho Pharma Co., Ltd. AM has received research grants from AbbVie, Asahi Kasei Pharma, Chugai Pharmaceutical Co., Ltd., Ono Pharmaceutical and speaker fees from Eli Lilly, AbbVie, Ono Pharmaceutical, Pfizer, Astellas Pharmaceutical, and Chugai Pharmaceutical Co., Ltd. MH received a research grant and/or speaker’s fees from Bristol-Myers, Eisai, Eli Lilly, and Mitsubishi Tanabe Pharma.

The remaining authors declare that the research was conducted in the absence of any commercial or financial relationships that could be construed as a potential conflict of interest.

The authors declare that this study received funding from Astellas Pharma Inc. The funder had the following involvement with the study: approval of the submission for publication.

## Publisher’s Note

All claims expressed in this article are solely those of the authors and do not necessarily represent those of their affiliated organizations, or those of the publisher, the editors and the reviewers. Any product that may be evaluated in this article, or claim that may be made by its manufacturer, is not guaranteed or endorsed by the publisher.
