# Supplementary material for: Dynamics of Type I and Type II Interferon Signature Determines Responsiveness to Anti-TNF Therapy in Rheumatoid Arthritis
Source: Front Immunol. 2022 Jun 6;13:901437. doi: 10.3389/fimmu.2022.901437 (PMC9208293; doi:10.3389/fimmu.2022.901437)
Supplement: Supplementary file 2 [file DataSheet_2.docx]

Supplementary Note

**Scripts which were used in single cell analysis.**

library(dplyr)

library(Seurat)

library(SeuratData)

library(patchwork)

pbmc.data <- Read10X_h5("GSM4819747_RA_filtered_feature_bc_matrix.h5")

pbmc <- CreateSeuratObject(counts = pbmc.data, project = "RA", min.cells = 3, min.features = 200)

pbmc[["percent.mt"]] <- PercentageFeatureSet(pbmc, pattern = "^MT-")

VlnPlot(pbmc, features = c("nFeature_RNA", "nCount_RNA", "percent.mt"), ncol = 3)

##Cell QC

pbmc <- subset(pbmc, subset = nFeature_RNA > 200 & nFeature_RNA < 5000 & percent.mt < 20)

#Normalization

pbmc <- NormalizeData(pbmc, normalization.method = "LogNormalize", scale.factor = 10000)

pbmc <- FindVariableFeatures(pbmc, selection.method = "vst", nfeatures = 2000)

all.genes <- rownames(pbmc)

pbmc <- ScaleData(pbmc, features = all.genes)

#Dimention reduction

pbmc <- RunPCA(pbmc, features = VariableFeatures(object = pbmc))

pbmc <- JackStraw(pbmc, num.replicate = 100)

pbmc <- ScoreJackStraw(pbmc, dims = 1:20)

JackStrawPlot(pbmc, dims = 1:15)

pbmc <- FindNeighbors(pbmc, dims = 1:10)

pbmc <- FindClusters(pbmc, resolution = 0.5)

pbmc <- RunUMAP(pbmc, dims = 1:20)

table(Idents(pbmc))

#Filter cluster with low number of cells

table(Idents(pbmc))

pbmc <- subset(pbmc, subset=seurat_clusters %in% c(1,2,3,4,5,6,7,8,9))

#Assigning cell type using canonical markers

#T

VlnPlot(pbmc, features = c("CD3D"))

#CD8

VlnPlot(pbmc, features = c("CD8A"))

#CD4

VlnPlot(pbmc, features = c("IL7R","CCR7"))

#B

VlnPlot(pbmc, features = c("MS4A1"))

#NK

VlnPlot(pbmc, features = c("GNLY","NKG7"))

#Monocyte

VlnPlot(pbmc, features = c("CD14","LYZ"))

pbmc <- RenameIdents(pbmc, `0` = "CD8", `1` = "CD8", `2` = "CD4",`3` = "CD4", `4` = "Mono", `5` = "NK", `6` = "B", `7` = "B", `8` = "Mono", `9` = "CD4")

#Define genes belong to type I IFN stimulated genes

responseIFNalpha<-c("ADAR","IFITM3","IFITM2","PYHIN1","PDE12","GAS6","GATA3","LAMP3","KLHL20","IFIT2","IFIT3","IFNAR1","IFNAR2","MX2","AXL","EIF2AK2","TROVE2","STAR","BST2","TPR","IFITM1")

responseIFNbeta<-c("IFITM3","IFITM2","TREX1","PYHIN1","HTRA2","UBE2K","TMEM173","IFI16","IFNAR2","IRGM","IRF1","MNDA","NDUFA13","PLSCR1","XAF1","C19orf66","STAT1","BST2","TLR3","IRG1","UBE2G2","CAPN2","IFITM1","PNPT1","AIM2","IKBKE","CDC34")

responseT1IFN<-unique(append(responseIFNalpha,responseIFNbeta))

pbmc<-MetaFeature(object=pbmc,features=responseT1IFN,meta.name="responseT1IFN",assay="RNA",slot="data")

VlnPlot(pbmc,features=c("responseT1IFN"),y.max=0.00032)

##Association test

test=data.frame(pbmc@meta.data)

test2=data.frame(pbmc@active.ident)

test3=cbind(test,test2)

CD4=test3[test3$pbmc.active.ident=="CD4",]$responseT1IFN

CD8=test3[test3$pbmc.active.ident=="CD8",]$responseT1IFN

Mono=test3[test3$pbmc.active.ident=="Mono",]$responseT1IFN

NK=test3[test3$pbmc.active.ident=="NK",]$responseT1IFN

B=test3[test3$pbmc.active.ident=="B",]$responseT1IFN

wilcox.test(Mono,CD4)

wilcox.test(Mono,CD8)

wilcox.test(Mono,NK)

wilcox.test(Mono,B)
