# Supplementary figures and images for: Dynamics of Type I and Type II Interferon Signature Determines Responsiveness to Anti-TNF Therapy in Rheumatoid Arthritis
Source: Front Immunol. 2022 Jun 6;13:901437. doi: 10.3389/fimmu.2022.901437 (PMC9208293; doi:10.3389/fimmu.2022.901437)

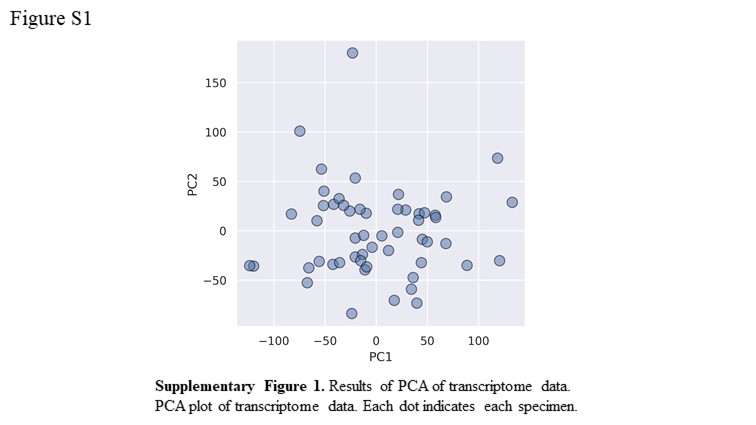

Supplement: Supplementary file 3 [file Image_1.jpeg]

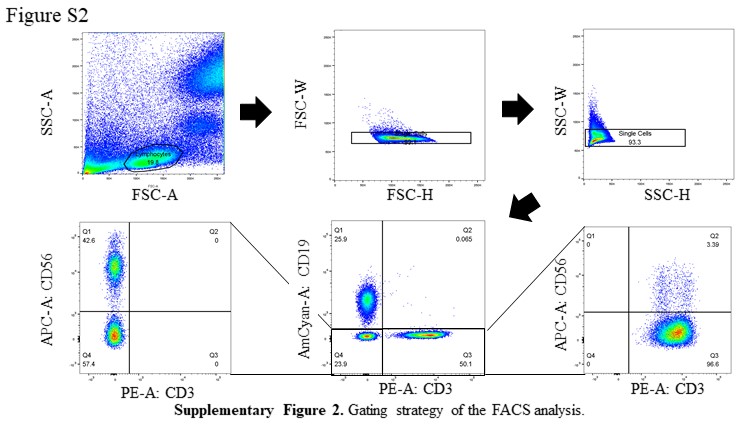

Supplement: Supplementary file 4 [file Image_2.jpeg]

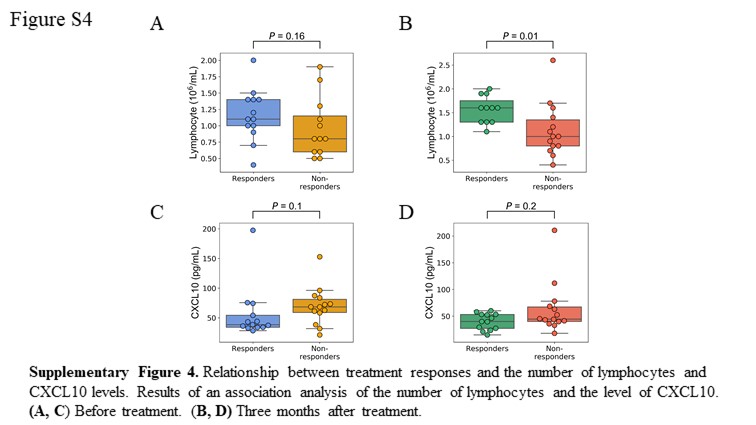

Supplement: Supplementary file 5 [file Image_3.jpeg]

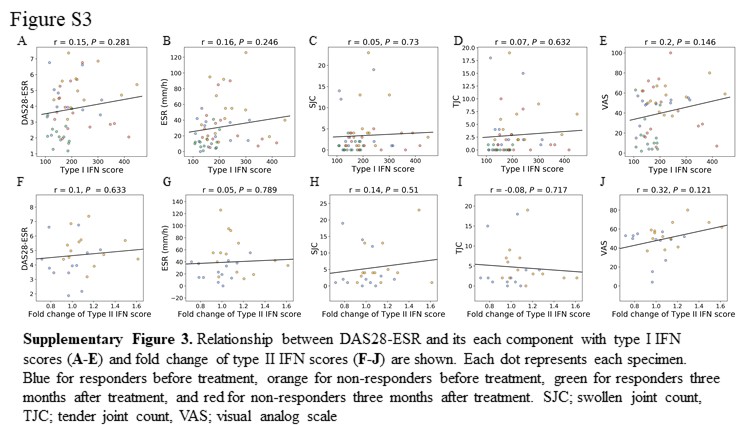

Supplement: Supplementary file 6 [file Image_4.jpeg]

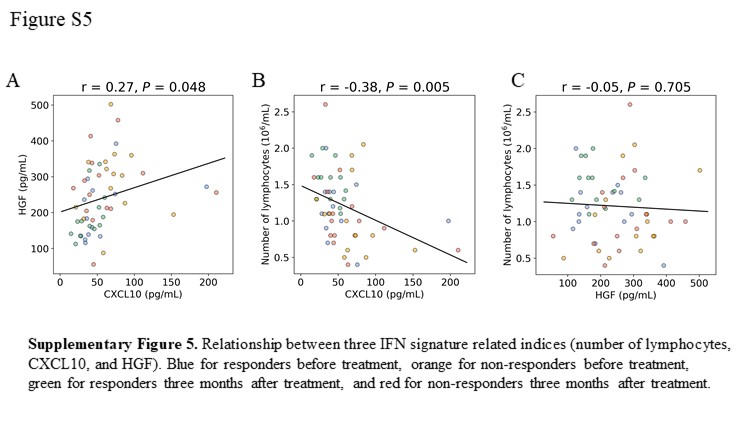

Supplement: Supplementary file 7 [file Image_5.jpeg]
